# Working Memory in ALS Patients: Preserved Performance but Marked Changes in Underlying Neuronal Networks

**DOI:** 10.1371/journal.pone.0071973

**Published:** 2013-08-08

**Authors:** Tino Zaehle, Andreas Becke, Nicole Naue, Judith Machts, Susanne Abdulla, Susanne Petri, Katja Kollewe, Reinhard Dengler, Hans-Jochen Heinze, Stefan Vielhaber, Notger G. Müller

**Affiliations:** 1 Department of Neurology, Medical School, Otto-von-Guericke-University, Magdeburg, Germany; 2 Leibniz-Institute for Neurobiology, Magdeburg, Germany; 3 German Center for Neurodegenerative Diseases, Magdeburg, Germany; 4 Department of Neurology, Medical School Hannover, Hannover, Germany; 5 Department of Psychiatry, Magdeburg Hospital GmbH, Magdeburg, Germany; Inserm, France

## Abstract

Amyotrophic lateral sclerosis (ALS) is a progressive neurodegenerative disease which affects the motor system but also other frontal brain regions. In this study we investigated changes in functional neuronal networks including posterior brain regions that are not directly affected by the neurodegenerative process. To this end, we analyzed the contralateral delay activity (CDA), an ERP component considered an online marker of memory storage in posterior cortex, while 23 ALS patients and their controls performed a delayed-matching-to-sample working memory (WM) task. The task required encoding of stimuli in the cued hemifield whilst ignoring stimuli in the other hemifield. Despite their unimpaired behavioral performance patients displayed several changes in the neuronal markers of the memory processes. Their CDA amplitude was smaller; it showed less load-dependent modulation and lacked the reduction observed when controls performed the same task three months later. The smaller CDA in the patients could be attributed to more ipsilateral cortical activity which may indicate that ALS patients unnecessarily processed the irrelevant stimuli as well. The latter is presumably related to deterioration of the frontal cortex in the patient group which was indicated by slight deficits in tests of their executive functions that increased over time. The frontal pathology presumably affected their top-down control of memory storage in remote regions in the posterior brain. In sum, the present results demonstrate functional changes in neuronal networks, i.e. neuroplasticity, in ALS that go well beyond the known structural changes. They also show that at least in WM tasks, in which strategic top-down control demands are relatively low, the frontal deficit can be compensated for by intact low level processes in posterior brain regions.

## Introduction

Although cognitive deficits in ALS have been reported sporadically for almost 100 years (e.g. [1-3]), until recently most neurologists considered ALS a pure neurodegenerative disorder of the motor system, associated with muscular atrophy, spasticity, respiratory dysfunction and bulbar signs but spared cognitive skills. This is due to the fact that cognitive deficits in those >90% of ALS patients, who do not develop concomitant fronto-temporal dementia, are subtle and become evident only with sophisticated neuropsychological testing of mainly executive functions [4-10]. The finding of rather mild cognitive and behavioral deficits stands in sharp contrast to pathological [11] and functional imaging data [12-16] that - in addition to the motor system - routinely reveal marked changes mainly in frontal regions and the anterior cingulate gyrus in ALS patients.

The discrepancy between widespread neuropathological changes and relatively intact behavior led us to hypothesize that functional neuronal networks in ALS patients could be more strongly altered than their relatively normal cognitive performance might suggest. These network changes can be the consequences of both neurodegeneration and compensatory neuroplasticity. In order to assess these potential changes, we analyzed event-related potentials derived from EEG scalp recordings while ALS patients performed a working memory (WM) task. The task was chosen as working memory is a multicomponent function that involves many processes, among them low-level short term storage and top-down strategic control [17,18]. As such it relies on a widely distributed neuronal network and, therefore, qualifies as a sensitive screening instrument in order to reveal functional network changes across many parts of the brain. The latter is particularly true since ALS patients perform normally in a wide range of routine neuropsychological tests of working memory, e.g. digit span [19]. Hence, it was not our primary aim to reveal behavioral deficits in the WM task chosen for this study. Firstly, this would not have reflected the usual finding of intact WM in these patients. Secondly, and even more important, the interpretation of differences in brain activity between patients and controls can be considered more reliable in the absence of group differences in behavioral performance that otherwise could have accounted for the observed effects in neurophysiology [20].

Within the neuronal WM network, the frontal cortex has been proposed to as act as a filtering set [21,22], that controls which information enters memory and is then stored in posterior brain regions like the parietal cortex [23]. Recently a potential electrophysiological online marker of the working memory storage process has been suggested, first by Vogel and Machizawa [24]. In their paradigm, a central arrow indicates whether stimuli presented in the left or in the right visual hemifield need to be encoded into memory while simultaneously presented stimuli in the other hemifield are to be ignored. After a short delay a test display is presented and subjects have to decide, for instance, whether the color of the test stimulus is identical to the color of one of the earlier stimuli (i.e., delayed-matching-to-sample). Electrophysiologically, during the delay phase a slow wave emerges that is more negative over posterior electrodes of the hemisphere contralateral to the task-relevant study items than over ipsilateral electrodes. Calculating the difference between contra- and ipsilateral waves results in the so-called contralateral delay activity (CDA). The more items have to be stored in memory, the more negative the CDA amplitude gets. However, the amplitude levels off when the number of presented items exceeds the individual working memory capacity (usually three to four items). Hence, the CDA can be considered a true online marker of visual short term memory [25], although which exact process within memory it reflects is not fully clear [26]. As such, the CDA has already been used before as an electrophysiological correlate of altered working memory in neurological patients, in this case Parkinson’s disease [10].

In the meantime, most fMRI [23,27-29] and MEG [30,31] studies have located visual short term memory storage into the intraparietal sulcus (IPS). Hence, the IPS can be considered the likely source of the CDA. Note, however, that the sensory areas also have been proposed to be involved in short term memory storage [32]. Furthermore, and in accordance with the above mentioned WM model, it has been shown that the CDA is under top-down control by the frontal cortex: Patients with unilateral prefrontal lesions lack the usual CDA load effect when the to-be-remembered stimuli are presented in the hemifield contralateral to their lesion [33]. This has been taken as evidence for disturbed frontal control of the storage process in posterior brain regions.

ALS is known to affect frontal brain regions more than posterior regions [11]. In this study we asked whether ALS patients nevertheless show alterations of a neurophysiological signature of the storage process in posterior cortex, namely the CDA component. Such a change would reveal remote effects on an intact brain region through compromised control by the frontal cortex. This finding would go beyond the demonstration of changes in a neuronal signature from a disease-affected region, an observation which would be somewhat trivial.

The progressive nature of the disease also raises questions regarding training effects. Usually, when accomplishing a task for the second time, one either performs better than before or needs less effort to perform at the same level. On a neuronal level, less activity in fewer brain regions is observed after a working memory task has been trained [34-36], whereby strengthened effective connectivity has been proposed to underlie this training effect [37]. With respect to the CDA it has been shown that in trials with distractors this component is reduced when subjects perform the WM task a second time. This has been interpreted as improved filtering ability, which, interestingly emerges whether subjects performed a dedicated training regimen in the meantime or not [38]. The situation is presumably different in patients with a progressive neurodegenerative disease where there is for example evidence of functional hyperactivity in areas that are structurally deteriorating [39]. Hence, when suffering from a progressive neurodegenerative disease it may be necessary to sustain or even increase neuronal activity to compensate the neuronal cell loss when the same task is performed once more later in the course of the disease. Therefore we compared ALS patients to healthy controls, similar in age and gender distribution, while they performed the lateralized working memory task at baseline and three months later. We used a variant of the original task by Vogel and Machizawa [24], in which subjects have to remember the colors of dots in either the left or right hemifield whereas the probe stimulus is presented at the center, a procedure which has been shown to increase the demands on working memory [40].

Our main hypotheses in this study were the following: 1) We assumed that working memory processes in ALS patients are altered despite their intact behavioral performance. These alterations should be reflected in modulations of the CDA component, a marker of working memory storage in posterior brain regions. 2) ALS patients presumably do not show the same training induced reductions of neuronal activity as controls because in the patients training effects are counteracted by a progressive neurodegenerative process.

EEG was recorded from 19 electrodes and ERPs time-locked to the onset of the study stimuli were calculated to assess the CDA amplitude.

## Methods

### Participants /Patients

Twenty three patients were recruited from the ALS outpatient clinics of the Departments of Neurology at the Medical School of the Otto-von-Guericke University Magdeburg and at the Medical School, Hannover. All recordings were performed at the DZNE Magdeburg. Patients were diagnosed according to the revised El Escorial criteria of the World Federation of Neurology [41]. All recruited patients (mean age: 58 years; range 33–82; 8 women) met the criteria for probable or definitive ALS as defined by the El Escorial diagnostic criteria for ALS [41]. Exclusion criteria included history of other neurological conditions that could affect motor performance and cognition (e.g. stroke, traumatic brain injury). The mean duration of illness was 26 months (range 4–77). Disease severity was assessed using the revised ALS Functional Rating Scale (ALSFRS-R; [42]), which assesses limb, bulbar and respiratory dysfunction. The mean ALSFRS-R score at the baseline visit was 38 (range 18–46) and at the second visit after three months 35 (range 15–46). This decline was statistically significant (t=3.37, p=.003). It reflects the natural course of the disease and is in line with previous reports [43]. The patients also underwent an extensive neuropsychological assessment both at baseline and at the second testing session. In concordance with the literature, this assessment revealed slight executive deficits regarding word fluency which also worsened over time (Regensburg Word Fluency Test (RWT) session 1: mean: 22.1, SD: 1.4; session 2: mean 19.6, SD: 1.3; t=2.79, p=.02). Other measures including those of WM, namely verbal digit span, were not found to be impaired and showed no decline over time (Wechsler Memory Scale-Revised (WMS-R) digit span I – forward: session 1: mean: 7.1, SD: 0.4; session 2: mean: 7.2, SD: 0.4; t=-0.4, p=.67; WMS-R digit span II – backward: session 1: mean: 6.45, SD: 0.3; session 2: mean: 6.43, SD: 0.5; t= 0, p=1).

All patients were taking the standard medication for ALS, Riluzole, which by means of its assumed neuroprotective properties has been shown to slow down the neurodegenerative process. Hence, withdrawal of this medication would have been unethical. The patients were taking no other substances that could interact with the central nervous system like Baclofen (against muscle spasticity) or antidepressants. Twenty three healthy individuals similar to the patients in age (mean age: 63 years, range 40–77) and gender distribution (9 women) were recruited as controls (p > 0.1). Ethical approval for all procedures was obtained prior to the study from the ethics committee of the University Clinic Magdeburg (11/06-75/11) and all participants gave written informed consent before participation.

### Working memory task

**Figure 1:**
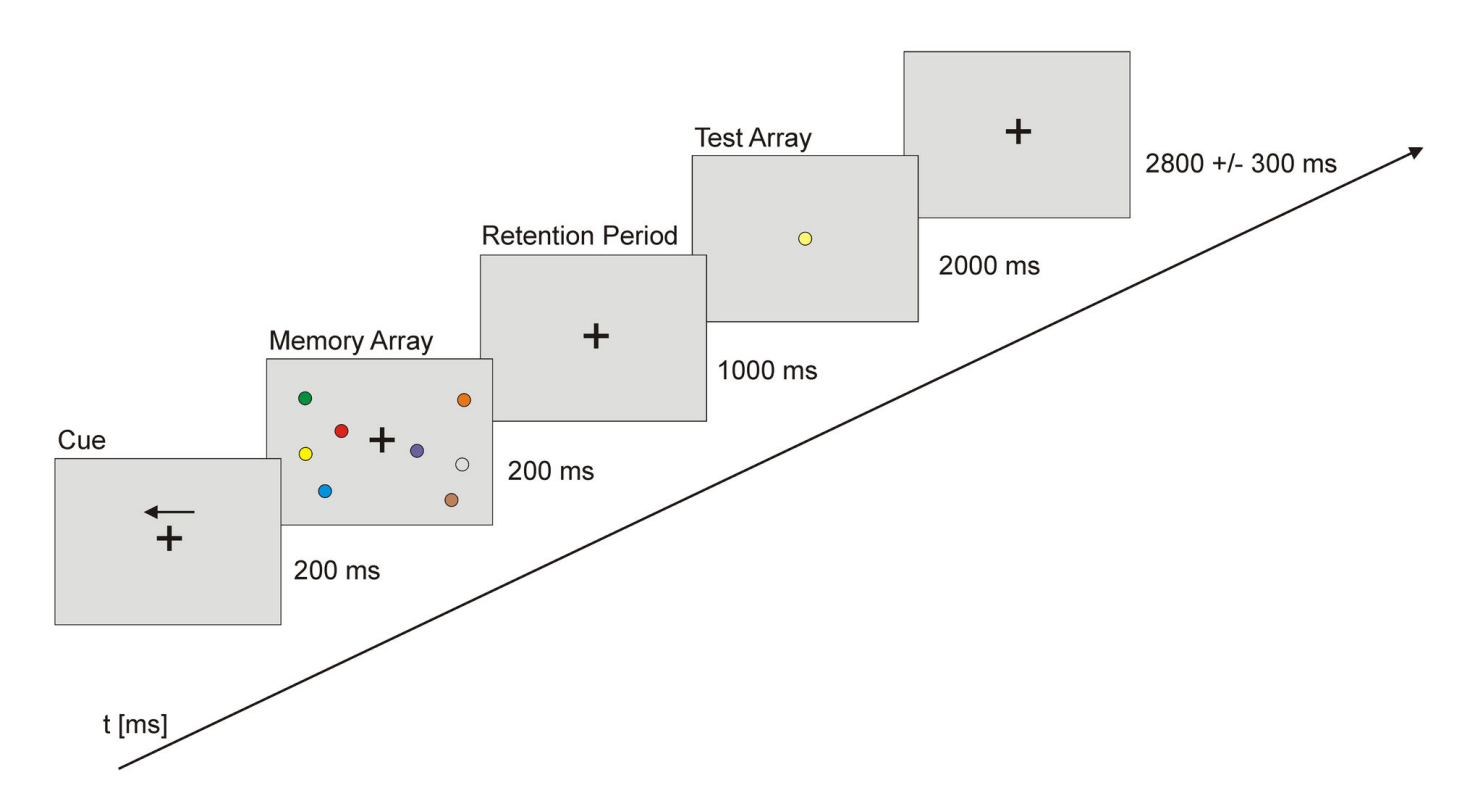
Schematic presentation of the paradigm. Subjects had to memorize the colors of the circles presented in the cued visual hemifield. After a delay they had to decide whether the single test circle’s color matched that of one of the earlier stimuli. Memory load varied between two and four colors that had to be kept in mind.

All participants performed a delayed matching-to-sample visuo-spatial WM task with concurrent EEG recording twice, with a delay of 3 months. Stimulus presentation was controlled by the Presentation software (Neurobehavioral Systems, USA). During each trial, subjects were presented with a fixation cross (2800 ± 300 ms) followed by an arrow (200 ms) indicating the hemifield (left/right) to be attended. A memory array was then presented within two rectangular regions that were centered to the left and right on a grey background. These two rectangular regions consisted of four (high load condition) or two (low load condition) colored circles (0.69°) with randomized position. The circles were randomly colored (blue, brown, green, red, cyan, yellow, orange, pink, black, white) whereby all presented circles had different colors in every trial. The memory array appeared for 200 ms and was followed by a retention period of 1000 ms during which subjects had to retain the memory array. This was followed by the presentation of a test array with one circle in the center of the screen, which was either identical or different in color compared to the circles shown in the memory array (cf. Figure 1). The test array was shown for max. 2000 ms. Within this time period participants had to make a push-button response to indicate whether the probe stimulus in the test array was identical in color to one of the stimuli in the memory array, which was the case in 50% of the trials. A session comprised 160 trials per load condition presented in pseudorandomized order and separated into four runs.

To assess the individual working memory performance, percent correct responses and reaction times were calculated and analyzed using a planned 2 x 2 x 2 mixed ANOVA with the within-subject factors *load* (low/high) and *session* (first, second) and the between subject factor *group* (patients, controls). Greenhouse–Geisser correction was applied in case of violation of the sphericity assumption.

### EEG recording and analysis

During the WM task, EEG was recorded from 19 standard scalp locations according to the European 10-20 system (Fp1, Fp2, F3, F4, F7, F8, Fz, Cz, C3, C4, T3, T4, Pz, P3, P4, T5, T6, O1, O2) using Ag/AgCl electrodes mounted in an elastic cap (Soft Cap EEGH-Z-*, Walter Graphtec GmbH). The vertical and horizontal electrooculogram was recorded with one electrode placed below and one placed approximately 1 cm to the external canthus of the right eye. EEG data were recorded by a PL-351 amplifier and the corresponding software (Walter Graphtek GmbH) referenced to electrode POz and sampled at 500 Hz. Impedances were kept below 10 kΩ. EEG preprocessing and data analysis were carried out in Brain Vision Analyzer 2.0 (Brain Products, Munich, Germany). EEG data were off-line filtered from 0.1 to 40 Hz and re-referenced to a common average reference. Event Related Potentials (ERPs) were segmented into 1500 ms epochs starting 300 ms before the onset of the memory array and covered the retention period. Baseline correction was accomplished between -300 ms and -200 ms. Segments containing ocular artifacts, movement artifacts, or amplifier saturation were excluded from the averaged ERP waveforms.

The CDA was measured at posterior parietal electrodes (P3/P4) as the difference between the ipsilateral and contralateral ERP waveforms. To test for specific alterations of the CDA in the course of ALS disease, we analyzed mean amplitudes for the CDA window (400–900 ms) with a planned mixed ANOVA with the within-subject factors *load* (low, high) and *session* (first, second) and the between subject factor *group* (patients, controls). Subsequently, post-hoc t-statistics were applied when appropriate.

## Results

### Behavior

**Figure 2:**
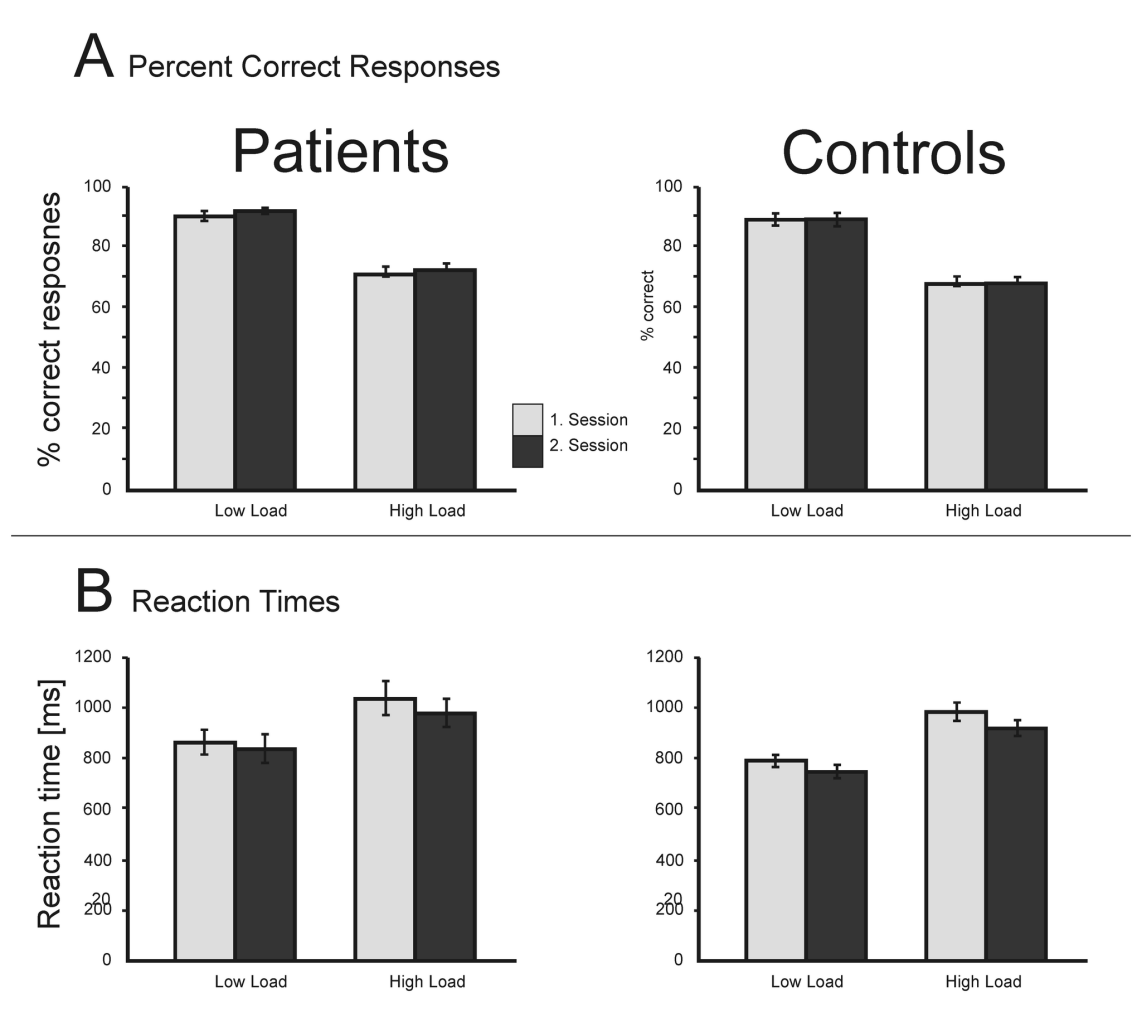
Behavioral data for the patients and their controls. Both groups made more errors and responded slower in the high than the low load condition and both groups were slightly faster in the second session. No group differences were observed in any condition.

For the percent correct responses the mixed ANOVA with the within-subject factors *load* (low, high) and *session* (first, second) and the between subject factor *group* (patients, controls) revealed a significant main effect for the factor *load* (F(1,44)=429.8, p<.001). Hence, both, patients and controls, showed the typical load effect with higher error rates in the high load compared to the low load condition. No other main effects (*session* (F(1,44)= 1.04, p=.31); *group* (F(1,44)=1.72, p=.19)) or interactions were significant. Thus, patients’ performance was unimpaired relative to that of controls. Training, i.e. performing the task a second time after 3 months, did not change the accuracy rates (see Figure 2).

For the reaction times, the ANOVA revealed a significant main effect of the factor *load* (F(1,44)=331.8, p<.001) and *session* (F(1,44)=10.7, p<.01). This demonstrates that all participants responded slower in the high load than in the low load condition and became faster in the second experimental session. Again there was no significant main effect of *group* (F(1,44)=1.26, p=.27) or any significant interaction.

### ERPs / CDA

**Figure 3:**
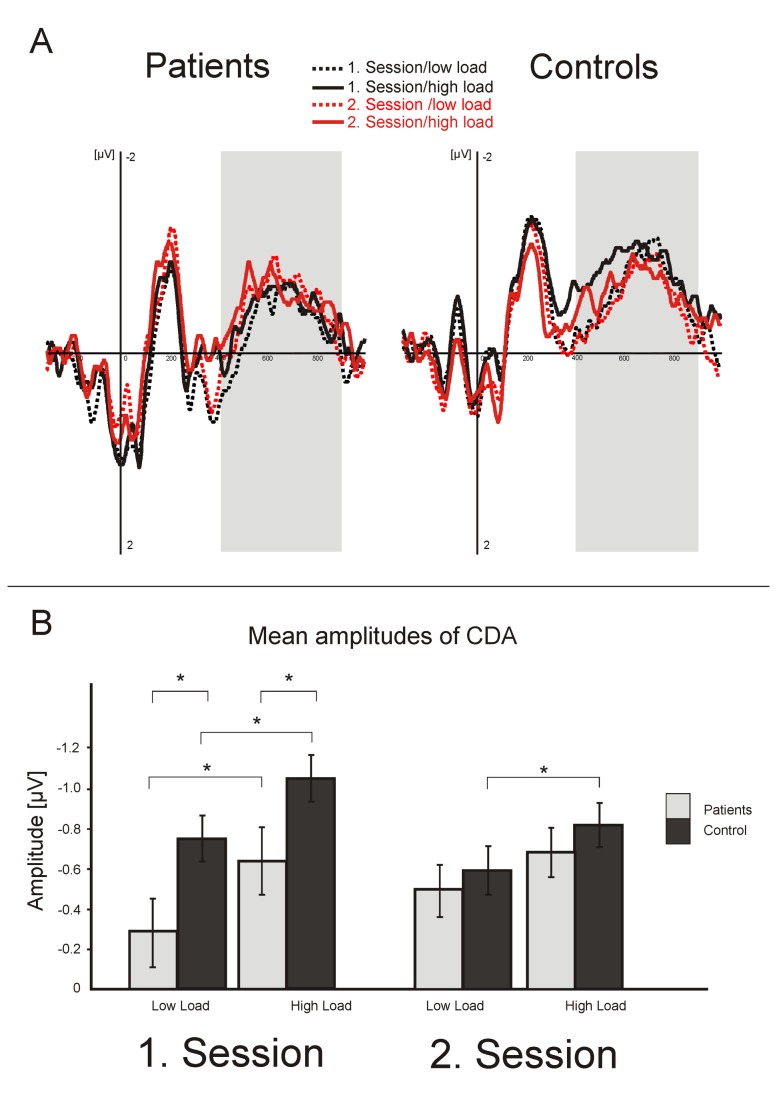
(a+b): The CDA component (original data in a, mean values in b) as depicted from electrodes P3/4. Both groups showed the typical load effect, i.e. higher (more negative) amplitudes in the higher load condition. In all conditions, patients displayed smaller CDA amplitudes than controls. Grey background color in a) marks the time window used for statistical analyses of the CDA.

The mixed ANOVA with the within-subject factors *load* (low, high) and *session* (first, second) and a between subject factor *group* (patients, controls) on the mean amplitude of the CDA revealed significant main effects for the factor *load* (F(1,44)=6.59, p=.01) demonstrating higher CDA amplitudes in the high load as compared to the low load condition, *group* (F(1,44)=5.1, p=.03) because of lower CDA amplitudes for patients than for controls, and a significant group x session interaction (F(1,44)=6.59, p=.01) due to lower CDA amplitudes for ALS patients for the low load (t(44)=2.07, p=.04) and high load condition (t(44)=2.47, p=.02) in the first session only (cf. Figure 3).

**Figure 4:**
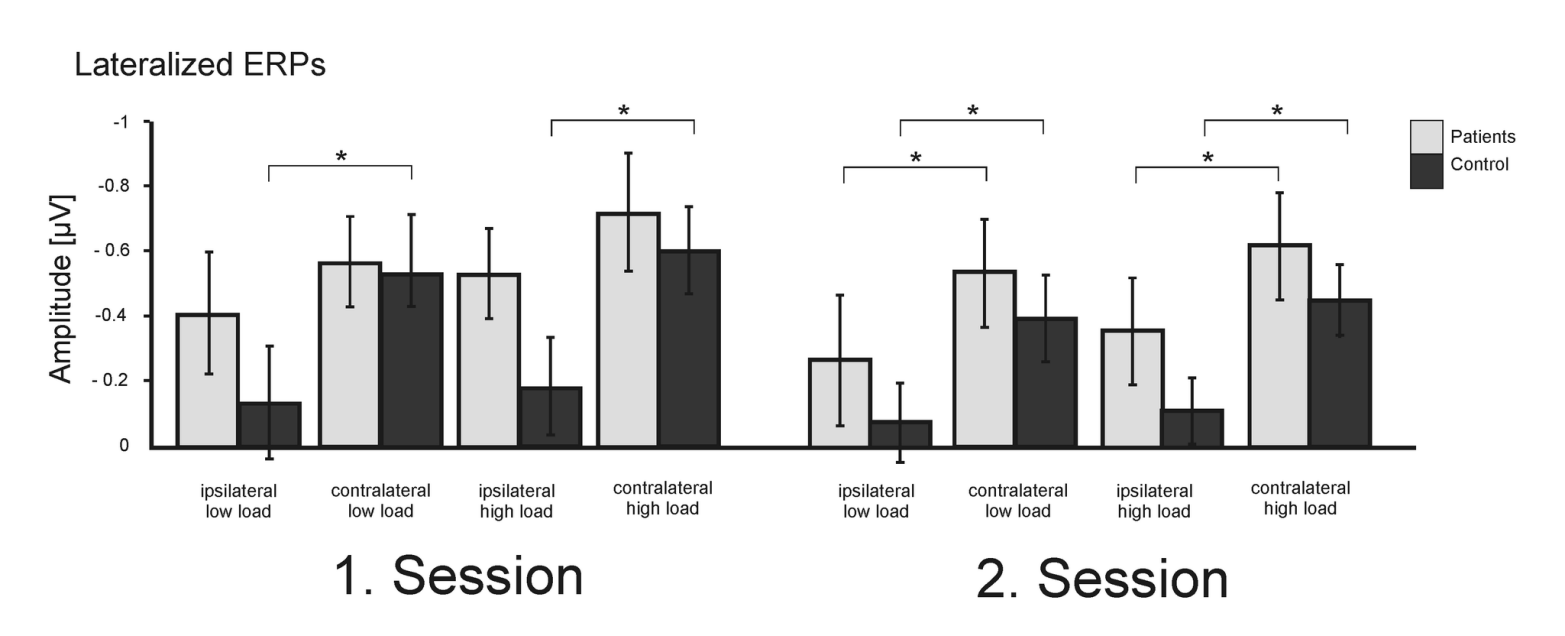
Mean slow wave amplitudes from electrodes ipsi- vs contralateral to the attended visual hemifield.

**Figure 5:**
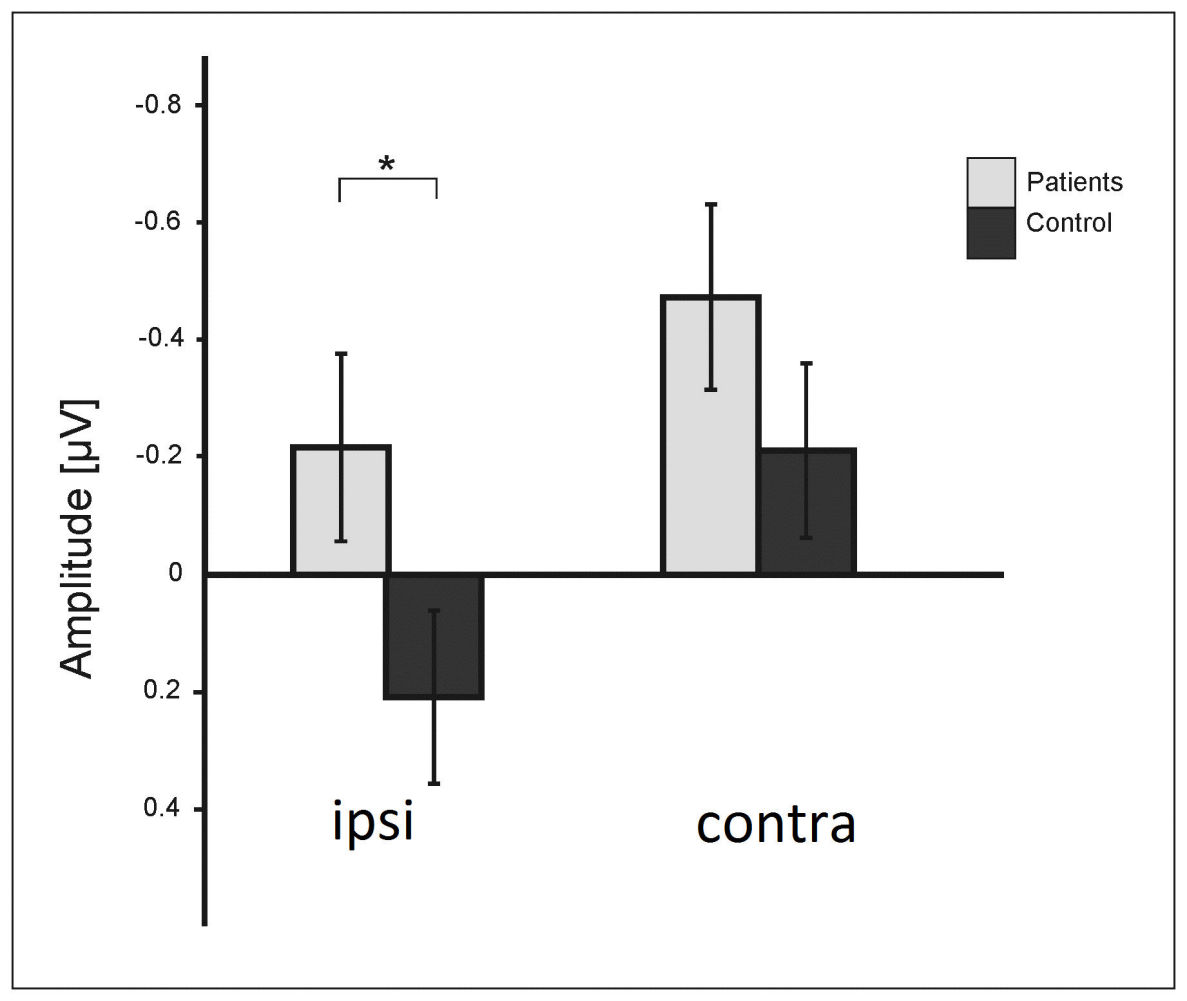
Lateralization effect, i.e. difference between ipsi- and contralateral ERP amplitudes. Ipsi- but not contralateral activity differed between patients and control subjects.

In order to explore the differences in CDA responses further, in a next step we analyzed the lateralized ERPs ipsi- and contralateral to the cued hemifield (cf. Figure 4) using a mixed ANOVA with the within-subject factors *load* (low, high), *session* (first, second), and *hemisphere* (ipsi/contra) and a between subject factor *group* (patients, controls). This analysis revealed significant main effects for the factor *hemisphere* (F(1,44)=80.3, p<.001) related to higher ERP amplitudes on the contralateral side, and a statistical trend for the factor *load* (F(1,44)=2.87, p=.08) indicating higher ERP amplitudes in the high load condition. Furthermore, the ANOVA revealed a significant group x hemisphere interaction (F(1,44)=5.1, p=.03) due to fact that the patient group generated significantly more ipsilateral activity than the control group (t(44)=-1.97, p=.05) with no differences on the contralateral side (t(44)=-1.26, p=.2) (cf. Figure 5). There was a significant *load x hemisphere* interaction (F(1,44)=6.65, p=.02) confirming the load dependency of the CDA. Finally, the session x group x hemisphere interaction (F(1,44)=6.05, p=.02) reveals that the patients generated significant more ipsilateral activity than the controls in the high load (session 1 t(44) =-2.34, p=.02; session 2 t(44) =-1.92, p=.06) but not in the low load condition (session 1 t(44) =-1.33, p=.2; session 2 t(44) =-1.28, p=.2). Contralateral activity did not differ between the groups, neither in the low load (session 1 t(44) =-0.47, p=.64; session 2 t(44) =-1.1, p=.28), nor in the high load condition (session 1 t(44) =-1.45, p=.14; session 2 t(44) =-1.5, p=.2).

## Discussion

In this study we assessed an electrophysiological signature of working memory storage, namely the CDA component, in 23 ALS patients and 23 healthy controls in order to reveal potential network changes related to neurodegeneration, but also compensatory neuroplasticity in the patients.

The ALS patients did not show any behavioral deficit in our task which required encoding of stimuli presented in the cued hemifield whilst ignoring stimuli in the other hemifield. They also performed normally in standard tests of working memory (digit span), a finding well in line with previous reports [19]. In contrast to their normal appearing performance, we observed several differences in the electrophysiological measures of the underlying WM processes in the ALS patients: Their CDA amplitudes were lower because of larger ipsilateral activity, i.e. they showed less laterality during working memory storage; their CDA load effect disappeared in the later recording session whereas controls generated altogether lower slow wave amplitudes when the experiment was repeated three months later a finding which is supported by a previous report [38]. As these modulations were not associated with behavioral deficits, it can be assumed that they reflect true network changes, as they cannot be attributed to performance differences between groups. Furthermore, as the CDA reflects the difference between attended and non-attended hemifield with sensory input from both fields being balanced, group differences in CDA amplitudes cannot be attributed to trivial factors like differences in scull thickness etc. This is underscored further by the finding that the slow waves over contralateral electrodes were identical in patients and controls. Hence, the CDA difference was obviously not driven by the hemisphere processing the relevant stimuli but resulted from larger ipsilateral activity in the patients.

The contribution of ipsilateral neuronal activity to the CDA has been neglected in most studies so far. One exemption is the work by Arend and Zimmer [44] who showed that adding stimuli to the unattended hemifield can increase the negative slow waves over the corresponding hemisphere, i.e. the one that is ipsilateral to the relevant hemifield. However, the authors observed this signal increase only when the relevant task was easy to accomplish. When the relevant task was made harder by increasing the number of to-be-memorized stimuli in the relevant hemifield, adding additional stimuli to the irrelevant hemifield did not change the ipsilateral activity anymore. The authors took this as evidence that ipsilateral activity, rather than reflecting compensation through recruitment of additional resources to perform the relevant task, reflects unnecessary storage of the to-be-ignored stimuli. This unnecessary storage is only performed as long as the relevant task is easy enough so that sufficient resources are available.

Based on this finding, we suggest that the more pronounced and load dependent ipsilateral activity in our patients likewise reflects that they unnecessarily processed the irrelevant items, i.e. refrained from filtering out this information. We attribute this unnecessary processing to impaired top-down control by the frontal cortex. As no physiological assessment of neuronal integrity of the frontal lobes (e.g. MR volumetry) was performed in the present study, inferences regarding frontal involvement must remain somewhat speculative. However, both the literature on this issue [11-16] and the increasing deficits in test of executive functions observed in our patients support the idea of frontal impairment in the patient group. Indeed, it has been shown that another process that mainly affects prefrontal regions, namely normal aging, compromises the early phase of the CDA so that older adults pay more attention to irrelevant information as their inhibitory processes are delayed [45,46]. In yet another study on the influence of frontal brain regions on the CDA, it was demonstrated that patients with unilateral prefrontal lesions do not show a CDA load effect when they pay attention to stimuli in the hemifield contralateral to their brain lesion [33]. The authors of this study proposed that their finding was attributable to a lack of prefrontal top-down control on posterior regions that generate the CDA. We likewise suggest that the reduced CDA amplitude in our ALS patients is related to prefrontal dysfunction, with the consequence of “unnecessary” activation of posterior regions in the hemisphere ipsilateral to the to-be-attended stimuli. This assumption is underpinned further by the observation that the load-effect was reduced in the second testing session, performed three months later, where it can be assumed – based on the documented clinical deterioration - that additional frontal degeneration had taken place in the meantime. Other than that, the patients unlike their controls showed no overall amplitude reductions compared to the initial experimental session. That is, whereas the controls in the second session seemed to have needed less neuronal resources, presumably related to enhanced effective connectivity [37], the patients obviously required the same amount of neuronal resources in the second session to sustain their initial performance levels.

The crucial question remains why in spite of the electrophysiological differences, which we propose to reflect impaired frontal top-down control of the posterior storage system, the patients showed unimpaired behavioral performance. A very simple, mechanistic explanation would be that – as the contralateral negative slow wave had the same amplitude in both groups – they had the same amount of neuronal resources available for the relevant stimuli with enough reserve for the patients to also encode the irrelevant stimuli without detrimental consequences on the relevant task. Indeed, it has been shown that with an easy task, healthy subjects also tend to encode the irrelevant stimuli. In that case healthy subjects – like our patients - show an increase in ipsilateral activity when the number of irrelevant stimuli in the related hemifield increases [44]. In this respect it is noteworthy that our patients tended to perform above average in our neuropsychological test battery, i.e. they can be assumed to be high performing. In other words, these high-performing subjects could “afford” the unnecessary storage of the stimuli presented in the irrelevant hemifield.

Two other explanations have to be considered. The first would assume that the increased ipsilateral activity in the patients reflected a compensatory, plasticity-related mechanism aimed at maintaining performance levels in a demanding task. Although we cannot rule out this possibility based on our data alone, as argued before this explanation seems unlikely based on the findings of a previous study. This study indicated that ipsilateral activity actually decreases when the relevant task becomes more difficult [44]. The third explanation for spared WM performance despite impaired frontal control is offered by a model which proposes that WM consists of two components, namely low-level feature binding and top-down strategic control. In this model, the amount to which the two components contribute to successful performance is proposed to be task- and age-dependent [47,48]. For example, both children and elderly show less strategic control as their frontal lobes have not matured yet or have already deteriorated, respectively. They, nevertheless, perform normal in several WM tasks although they do not display CDA load effects in these tasks. This decoupling of CDA and observed behavior has been taken as evidence that in case a WM task imposes only low strategic control demands, low-level processes performed in posterior brain regions are sufficient to sustain working memory [49]. With WM tasks that put higher challenges on strategic control than the present one, like spatial n-back tasks, ALS patients have been demonstrated to perform below the levels of their age-matched controls [19].

In sum, the present results once more show, how flexible the human brain can compensate even for marked neuronal damage - given the underlying disease is slowly progressing. Because of largely intact posterior brain regions supporting low-level processes, ALS patients in this study showed normal WM performance despite of reduced frontal control as reflected by smaller CDA amplitudes.
